# Resting State Functional Connectivity Patterns Associate with Alcohol Use Disorder Characteristics: Insights from the Triple Network Model

**Published:** 2025-04-08

**Authors:** Daniel Guerrero, Mario Dzemidzic, Mahdi Moghaddam, Mintao Liu, Andrea Avena-Koenigsberger, Jaroslaw Harezlak, David A. Kareken, Martin H. Plawecki, Melissa A. Cyders, Joaquín Goñi

**Affiliations:** 1 Edwardson School of Industrial Engineering, Purdue University, West-Lafayette, IN, USA; 2 Purdue Institute of Integrative Neuroscience, Purdue University, West-Lafayette, IN, USA; 3 Department of Neurology, Indiana University School of Medicine, Indiana Alcohol Research Center, Indianapolis, IN, USA; 4 Department of Radiology and Imaging Sciences, Indiana University School of Medicine, Indianapolis, IN 46202, USA; 5 Center for Neuroimaging, Department of Radiology and Imaging Sciences, Indiana University School of Medicine, Indianapolis, IN 46202, USA; 6 Department of Epidemiology and Biostatistics, Indiana University Bloomington, Bloomington, IN, USA.; 7 Department of Psychiatry, Indiana University School of Medicine, Indianapolis, IN USA; 8 Department of Psychology, Indiana University Indianapolis, Indianapolis, IN, USA.; 9 Weldon School of Biomedical Engineering, Purdue University, West-Lafayette, IN, USA

## Abstract

Prolonged alcohol use results in neuroadaptations that mark more severe and treatment-resistant alcohol use. The goal of this study was to identify functional connectivity brain patterns underlying Alcohol Use Disorder (AUD)-related characteristics in fifty-five adults (31 female) who endorsed heavy alcohol use. We hypothesized that resting-state functional connectivity (rsFC) of the Salience (SN), Frontoparietal (FPN), and Default Mode (DMN) networks would reflect self-reported recent and lifetime alcohol use, laboratory-based alcohol seeking, urgency, and sociodemographic characteristics related to AUD. To test our hypothesis, we combined the triple network model (TNM) of psychopathology with a multivariate data-driven approach, regularized partial least squares (rPLS), to unfold concurrent functional connectivity (FC) patterns and their association with AUD characteristics. We observed three concurrent associations of interest: i) drinking and age-related cross communication between the SN and both the FPN and DMN; ii) family history density of AUD and urgency anticorrelations between the SN and FPN; and iii) alcohol seeking and sex-associated SN and DMN interactions. These findings demonstrate the utility of combining theory- and data-driven approaches to uncover associations between resting-state functional substrates and AUD-related characteristics that could aid in the identification, development, and testing of novel treatment targets across preclinical and clinical models.

## 1. Introduction

Alcohol use disorder (AUD) is a chronic relapsing brain disorder affecting over 28 million people in the United States alone (SAMHSA, 2023). Chronic alcohol use leads to neuroadaptations that result in decreased sensitivity to alcohol, continued drinking despite negative consequences, negative reinforcement consumption, and relapse (Koob & Volkow, 2010), all of which mark more severe and treatment-resistant alcohol use. As AUD progresses, brain networks implicated in alcohol use shift away from reward to circuits underlying decision making, negative affect, memory, and craving (Koob & Volkow, 2010). In this work, we combine theory- and data-driven approaches to identify underlying neural substrates and alterations of between- and within-network interactions that characterize known AUD-related characteristics known to impart risk for AUD.

Functional balance among neural networks is necessary for adaptive cognitive and behavioral function (Friston, 2011; Wang et al., 2021). The Triple Network Model (TNM) posits that the salience network (SN) mediates switching between the default mode (DMN) and frontoparietal (FPN) networks (V. Menon, 2011; Sridharan et al., 2008) to accomplish a balance between endogenously and exogenously driven mental activity. The FPN is responsible for high-level cognitive functions and goal-oriented tasks (Miller & Cohen, 2021), and comprises the lateral prefrontal cortex, anterior inferior parietal lobule, and middle frontal gyrus (Uddin et al., 2019). The SN and FPN are active during tasks, such as those requiring external cognitive demands (Fox et al., 2005; Seeley et al., 2007; Uddin & Menon, 2009). In contrast, the DMN is most active in absence of external task demands (Greicius et al., 2002; Raichle et al., 2001), and more implicated in internal mental processes (e.g., introspection, future planning, mind wandering). The DMN includes the ventral and dorsal medial prefrontal cortex, anterior and posterior cingulate as well as retrosplenial cortex, precuneus (mostly ventral), inferior parietal lobule, lateral temporal cortex, and hippocampal formation (Buckner et al., 2008). The SN plays a central role in balancing these (and other) functional networks by detecting and prioritizing sensory input to guide attention, attending to motivationally salient stimuli, and recruiting appropriate functional networks to modulate behavior (Peters et al., 2016). Under the TNM, cognitive and emotional dysfunction from psychopathology reflects disruption in the functional integration among these three networks, both at rest and during task (Elsayed M, 2024; B. Menon, 2019).

Alcohol exposure alters neural circuits and diminishes cognitive capacity (Fritz et al., 2019; Shokri-Kojori et al., 2017; Squeglia et al., 2014; Topiwala et al., 2022; Wagner et al., 2006; Zahr & Pfefferbaum, 2017) among other negative consequences. Within- and between-alterations in SN, FPN and DMN functional connectivity patterns are particularly important in AUD (Canessa et al., 2021; Chanraud et al., 2011; Elsayed M, 2024; Maleki et al., 2022; Suk et al., 2021). With greater frequency, quantity, and duration of drinking, neuroadaptations in the brain will further alter drinking behavior itself (Koob & Volkow, 2010; Greenfield et al., 2014; Nieto et al., 2021). More specifically, regions within the DMN that are normally deactivated during task processing in healthy controls exhibit the opposite pattern in individuals with AUD, reflecting dysregulation and compromised functional connectivity (Chanraud et al., 2011; Schulte et al., 2012). Similarly, dysregulation and decreased FPN connectivity during task execution (Maleki et al., 2022; Squeglia et al., 2014), as well as slow decision-making and abnormalities in SN circuits (Galandra et al., 2018; Suk et al., 2021), are common in those with AUD.

Multiple factors contribute to the onset and progression of AUD. Impulsivity is a key risk factor for AUD (Shin et al., 2012), particularly driven by emotionally provoked rash action (i.e., “urgency”; Cyders & Smith, 2008; Whiteside & Lynam, 2001; Zorrilla & Koob, 2019). It also contributes to greater drinking quantity and frequency over time (Littlefield et al., 2014), earlier onset of AUD (Dick et al., 2010), and to a worse treatment response (Hershberger et al., 2017; Whitt et al., 2019). With greater severity, the transition to AUD is theorized to reflect the change from impulsive to compulsive alcohol use. Compulsivity and urgency likely represent a shift from action-outcome to stimulus-response behaviors, resulting in within- and between-network changes in FPN, SN, and reward-related network interactions among others (Fan et al., 2017; Giuliano et al., 2019; Gürsel et al., 2018; O’Tousa & Grahame, 2014; Su et al., 2024), some of which are also evident in obsessive-compulsive disorder (Jones et al., 2023; Sripada et al., 2014). Those with a biological family history of AUD are at higher risk to develop AUD (Kareken et al., 2010; NIAAA, 1997; Nurnberger et al., 2004; Oberlin et al., 2013), with evidence suggesting altered transitions between cognitive states implicating altered functional networks related to TNM (Amico et al., 2020). Finally, males and younger individuals tend to engage in heavier drinking, although negative consequences for drinking are worse in females and, recently between male and female drinking and AUD-rates has narrowed (Keyes et al., 2010). While not specific to AUD, sex and age differences have been identified in various conditions implicating the FPN, SN, and DMN and/or their within- and across network connectivity (Cummings et al., 2020; de Dieu Uwisengeyimana et al., 2022; Ernst et al., 2019; Helpman et al., 2021; Lawrence et al., 2020).

In this study we investigated the multiple associations between AUD-related characteristics and their corresponding functional neural substrates, using the TNM framework (V. Menon, 2011) to assess how the FPN, SN, and DMN and their interactions relate to factors conferring risk for AUD. The model theorizes that the SN mediates communication between FPN and DMN and that dysregulation between these three networks leads to cognitive and emotional disorders (Figure 2A). TNM has a potential to better understand how AUD affects brain functionality (Elsayed M, 2024), as these three networks regulate and balance relationships between introspective states (such as those related to urges and internal visceral sensation) and directed attention to either external cognitive demands (V. Menon, 2011; Sridharan et al., 2008) or alcohol-related cues and phenomena (Suk et al., 2021).

We therefore hypothesized that top-down regulating mechanisms between the SN, FPN and DMN and their intrinsic functional connectivity patterns would be disrupted reflecting AUD symptoms, alcohol use, alcohol seeking, urgency, and family history of AUD. In this study, we applied a data-driven regularized partial least squares (rPLS) approach to elucidate how resting state functional connectivity related to these characteristics and to provide fine-grained description revealing specific regions contributing to these associations.

## 2. Methods

### 2.1 Participants information

Participants (n=55; 31 female, 33 white, mean age=32.18, SD=9.9) were healthy, community dwelling adults who reported current heavy alcohol use (average 21.20 drinks per week (SD=26.78) ), and who were recruited as part of a larger study (Garrison G. & Wu, 2025, under review). Participants were recruited to ensure both a range of lifetime drinking history and, for safety, sufficient recent experience with alcohol’s effects. Inclusion criteria included self-reported good health, aged 21–55, able to understand/complete questionnaires and procedures in English, body mass index between 18.5 and 32 kg/m^2^. Exclusion criteria for the current analysis included contraindications to imaging (e.g., metal in body, left-handedness), pregnancy or breast-feeding, desire to be treated for AUD or any substance use disorder or court ordered not to drink alcohol, medical/mental health conditions or medications that may influence data quality or participant safety, and evidence of substance intoxication (positive urine drug screen for amphetamines/methamphetamines, barbiturates, benzodiazepines, cocaine, opiates, or phencyclidine and associated alteration of vital signs or subjective assessment consistent with intoxication) or positive breath alcohol reading on arrival on any study day.

### 2.2 Participants measures

#### Demographics.

Participants self-reported their age, biological sex, race, ethnicity, and highest level of completed education. In addition, participants underwent evaluations for the following measures.

#### *The Semi-Structured Assessment of the Genetics of Alcoholism* (Bucholz et al., 1994).

We used the alcohol module of the SSAGA to estimate a lifetime diagnosis of DSM-5 AUD (2+ symptoms). Of the 55 participants in the sample, 34 (18 women, 16 men) met the criteria for AUD (61.8%), with 21 falling in the mild, 7 in the moderate, and 6 in the severe categories. The family history module was used to quantify the presence of AUD in first- and/or second-degree relatives for each participant. A Family history density (FHD) score (Stoltenberg & Dd, 1998) was calculated for each participant, based on the degree of biological relatedness, in which parents and full-siblings with a lifetime history of DSM-5 alcohol dependence contributed 0.5 for each person, each dependent grandparent or sibling of parents contributed 0.25, and non-affected biological relatives contributed zero. We calculated FHD as the sum of weights divided by the number of counted relatives.

#### *Timeline Follow-back of Alcohol Use* (TLFB; Sobell & Sobel, 1992).

Recent drinking was characterized by using the TLBF procedure to provoke asking participant recollection of how many drinks they had on any drinking occasion over the previous thirty-five days. The following variables were calculated: number of drinking days/week (TLFB_DDW_), average number of drinks/drinking day (TLFB_DDD_), and the greatest number of drinks consumed on any single drinking day (TLFB_GDD_). The Timeline Follow-back has been shown to produce valid measures of one’s recent drinking behaviors (Sobell & Sobel, 1992).

#### *Concordia Lifetime Drinking Questionnaire* (Chaikelson et al., 1994).

The Concordia scale is a self-report measure of total amount of alcohol drinking across the lifespan. Individuals report current and historical alcohol use, including age when alcohol use began, patterns of drinking (and changes in them), and quantity and frequency of drinking. Information is then summed to create several measurements, including the total amount of alcohol (assessed in kilograms) consumed over one’s lifetime to date (LDH_KG_), which is the score used in the current study. The scale was shown to provide reliable and valid information concerning one’s lifetime drinking (Chaikelson et al., 1994).

#### *The Short UPPS-P Impulsive Behavior Scale* (Cyders et al., 2014).

The Short UPPS-P is a 20-item self-report scale measuring five related, although distinct, tendencies toward rash action. Only the positive (Pur) and negative (Nur) urgency scales were used in this study. Items are asked using a four-point Likert scale from 0 (Agree Strongly) to 4 (Disagree Strongly). Items are reverse-scored and averaged so that higher scale scores reflect greater impulsive tendencies.

#### Alcohol Seeking.

Alcohol seeking was assessed using intravenous alcohol self-administration. The Computer-assisted Alcohol Infusion System software was used to compute alcohol infusion rates required for exposure control (Zimmermann et al., 2008, 2009). Infusion sessions began with a priming interval, during which participants’ breath alcohol concentration was increased to 60 mg/dL over 15 minutes and subsequently maintained for approximately 25 minutes to assess subjective and physiological sensitivity to alcohol. Following the priming interval, participants completed a 2.5-hour self-administration session, which included the Constant Attention Task (CAT; Plawecki et al., 2013) to earn alcohol or an alternative reinforcer (in this case, water) reward. The procedure required an escalating number of successful CAT trials to receive either of two rewards (each on independent schedule), with task difficulty adjusted to maintain ~50% success rate. Consistent with our prior work, alcohol seeking was quantified as cumulative work for alcohol or water (cwa and cww respectively), corresponding to the total number of trials each participant completed to earn alcohol or water infusion rewards (Plawecki et al., 2013, 2018). Alcohol seeking was conducted under two conditions: 1) neutral (N), and 2) aversive (A), with seeking in the presence of aversive sights and sounds (Garrison G. & Wu, 2025) modeling the transition to compulsive use.

### 2.3 General Procedures

Participants completed two intravenous alcohol self-administration sessions, one in which working for alcohol was paired with aversive stimuli and another pairing work with neutral stimuli, using a progressive ratio paradigm (see full methods and results from behavioral session in Clinicaltrials.Gov; Garrison G. & Wu, 2025), followed by a resting-state fMRI session. All sessions were conducted on separate days, with the fMRI session at least a week after the second infusion session (modal days = 7, median days = 13, mean days = 26.5).

### 2.4 Image acquisition

Imaging was conducted on a Siemens 3T Prisma (Erlangen, Germany) MRI scanner with a 64-channel head coil array. A high-resolution anatomical volume 3D Magnetization Prepared RApid Gradient Echo sequence (MPRAGE; Lifetime Human Connectome Protocol parameters: 1 slab with a 50% distribution factor, 208 sagittal slices/slab, slice oversampling 23.1%, 0.8 mm slice thickness, 256 mm field-of-view (FoV), 93.8% FoV phase, 320×320 matrix, repetition/echo/inversion time TR/TE/TI= 2400/2.22/1000 ms, flip angle= 8 deg, GRAPPA acceleration= 2, 0.8×0.8×0.8 mm^3^ voxels) was acquired first. Participants then completed a resting-state fMRI (rsfMRI) scan with an instruction to think about nothing in particular while fixating their gaze on a centrally located white crosshair shown on a black background once the scan began. This eight-minute blood oxygenation level dependent (BOLD) rsfMRI scan used a multi-band (MB) echo-planar imaging (EPI) sequence (Center for Magnetic Resonance Research at the University of Minnesota, gradient echo, 616 BOLD volumes, TR/TE= 780/29ms, flip angle 54 deg, field-of-view 220×220 mm^2^, matrix 88×88, fifty-five 2.5 mm thick slices, 2.5×2.5×2.5 mm^3^ voxel, slice acceleration factor= 5) (S. M. Smith et al., 2013). BOLD fMRI acquisition was preceded by a pair of phase-reversed spin echo field mapping scans (3 A-P and 3 P-A phase direction volumes, TR/TE= 1200/64.40 ms); other imaging parameters matched the rsfMRI acquisition.

### 2.5 Image Preprocessing

Preprocessing was completed with an in-house Bash and Python 3.6 based pipeline using FMRIB Software library (FSL version 6.0.1). T1-weighted MPRAGE image of each participant was denoised prior to brain masking and extraction with ANTs (Avants et al., 2009) and then nonlinearly transformed (FSL’s *flirt* and *fnirt*) to Montreal Neurological Institute (MNI) MNI152 standard space. This MNI-to-T1 transformation was followed by T1-to-EPI transformation (see EPI preprocessing) allowing standard-to-native (i.e., MNI-to-EPI) and inverse (i.e., EPI-to-MNI) transformations required to apply standard space atlases. rsfMRI data were processed in native BOLD EPI space of each participant. The preprocessing steps included BOLD volume distortion correction using FSL’s *topup/applytopup* (utilizing phase-reversed spin echo field mapping scans), head motion realignment (*mcflirt*), T1-to-EPI registration (linear, nonlinear, and boundary-based registrations), normalization to mode 1000, and spatial smoothing with a 6 mm isotropic full width at half maximum (FWHM) Gaussian kernel.

Following recommendations for robust preprocessing (Eklund et al., 2016), the preprocessed data were entered into FSL’s MELODIC (Nickerson et al., 2017) for independent components analysis (ICA)-based denoising with ICA-AROMA (Pruim, Mennes, Buitelaar, et al., 2015; Pruim, Mennes, van Rooij, et al., 2015). A single step regression was applied to the denoised BOLD volumes to avoid reintroducing artifacts in the preprocessed denoised data (Lindquist et al., 2019; Phạm et al., 2023). Specifically, regressors were applied that 1) indexed head motion using the realignment and their derivatives (Power et al., 2015), 2) accounted for physiological noise (first five signals obtained by PCA from the white matter and cerebrospinal fluid-eroded masks; an implementation of aCompCor (Muschelli et al., 2014), 3) performed high-pass filtering (f_min_ = 0.009 Hz) using Discrete Cosine Transforms bases (Shirer et al., 2015), and 4) included outlier volume despiking (Phạm et al., 2023). The outliers were determined using the significant “DVARS” metrics obtained on the single-regression preprocessed data as described in (Phạm et al., 2023). This procedure tagged a mean of 1.31% (Standard Deviation: 1.24%; range: 0 – 7.29%) of residual high head motion volumes across all participants.

Individual-level rsFC matrices were determined by pairwise Pearson correlation coefficients of mean regional BOLD time-series. We implemented the Schaefer 300 cortical parcellation (Schaefer et al., 2018) and the 32-region Scale II Melbourne Subcortical Atlas (Tian et al., 2020) to assess functional connectivity among 332 brain region pairs (cerebellum excluded). To facilitate the interpretation, we aggregated the cortical regions into seventeen resting-state functional networks (RSNs) as proposed by Yeo (Thomas Yeo et al., 2011).

### 2.6 Selection and processing of participant phenotypes.

As the TLFB and lifetime drinking history variables were skewed, with long positive tails, they were logarithmically transformed (see Figure S3). The behavioral alcohol seeking variables were transformed to capture the contrast between working for alcohol and water in the neutral and aversive sessions and termed alcohol preference (ap; see Figure S1):

$$neutral\text{\_}ap=n_{-}cwa-n_{-}cww\;aversive\text{\_}ap=a_{-}cwa-a_{-}cww$$

All variables were then Z-scored to standardize the magnitudes among variables presented to the subsequent learning algorithms. We then applied Principal Component Analysis (PCA) to project these AUD-related characteristics into a new set of orthogonal variables. Only PCA components/latent variables with eigenvalues greater than one were preserved and analyzed. Positive and negative urgency were compressed into a single urgency variable reflecting disposition to rash action in response to emotion, regardless of the valence (75% explained variance) and labeled “Urgency”. Drinking history variables were compressed into one component that reflected a combination of recent and lifetime consumption patterns (70% explained variance) and labeled “Drinking.” Alcohol seeking was further compressed into a single behavioral variable that encodes the general willingness to seek alcohol across both sessions (74% explained variance, which happened to be the mean value of neutral_ap and aversive_ap) and labeled “Alcohol seeking” (see Figures S2 and S4). After these preparatory steps, the final set of participant characteristics included eight representative variables: sex (male/female), age (in years), education (in years), FHD, AUD symptoms, Drinking, Urgency, and Alcohol seeking (Table 2; see Figure S5).

### 2.7 Partial Least Squares

We used partial least squares (PLS) analysis (Krishnan et al., 2011; McIntosh & Lobaugh, 2004; Wold, 1966) to identify associations between rsFC patterns and participant phenotypic characteristics (see Table 2). PLS is an unsupervised method designed to find intrinsic relations between two sets of multidimensional variables (modalities). It can be considered an extension of linear regression models to handle multidimensional variables both in the covariate and the response (Wold, 1966). PLS identifies hidden associations between data domains by projecting both modalities into a new space and finding linear combinations that maximize the covariance between them. The associations are captured in a set of orthogonal components describing how the variables interact between domains.

PLS models linear relationships between two sets of variables X and Y, covariates and responses respectively. It assumes that X and Y can be linearly decomposed as:

$$X=\Phi P+E_{X}$$

$$Y=\Psi C+E_{Y}$$

Where Φ and Ψ are latent vectors of X and Y respectively, P and C are the coefficients associated with each variable, and $E_{X}$ and $E_{Y}$ are error terms.

**Figure 1.**
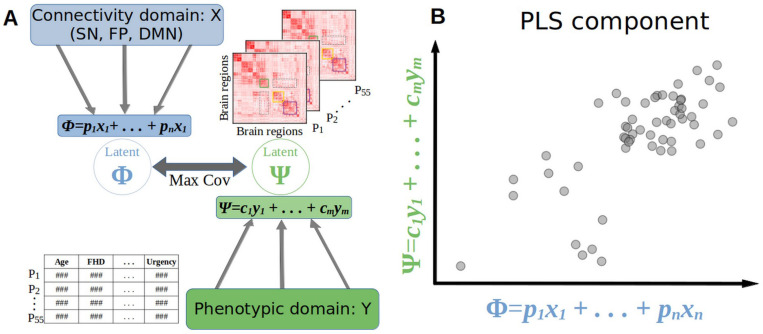
A) Schematic representation of the Partial Least Squares (PLS) analysis. Two sets of variables (here connectivity and phenotypic domains) are projected to a latent space of lower dimensionality (X and Y, respectively). For each PLS component, a linear regression is performed in the new space and the linear combinations are optimized to maximize the covariance between the variables in the latent space. B) Example of a PLS component maximizing covariance between the two domains (axes are the corresponding latent variables).

The coefficient matrices P and C are set as to maximize the covariance between the latent vectors Φ and Ψ. (see Figure 1).


$$\Phi =XP^{-1}$$

$$\Psi =YC^{-1}$$

$$max_{\left\{P,C\right\}}COV(\Phi ,\Psi )$$

In addition to handling multidimensionality in both modalities, PLS has been successfully applied in diverse biological settings where the number of variables/features is considerably larger than the sample number— for example, bioinformatics, various “omics”, and chemometrics (Chung & Keles, 2010; Krishnan et al., 2011; Land et al., 2011; Mehmood & Ahmed, 2016; Yoshida et al., 2017). Hence, PLS is well suited for brain connectomic analyses (Fornito et al., 2016; Sporns, 2011), where the number of brain regions is generally larger than the sample size (number of participants), and the subsequent number of connections between regions increases quadratically (Krishnan et al., 2011; McIntosh & Lobaugh, 2004).

**Figure 2.**
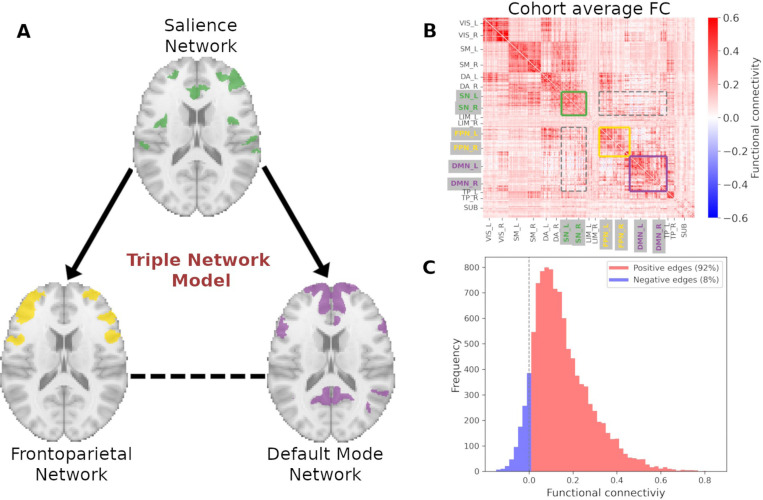
Functional connectivity within and between Frontoparietal (FPN), Default Mode (DMN), and Salience (SN) networks using a 300-region cortical parcellation (Schaefer et al., 2018) and a 17-network functional atlas (Thomas Yeo et al., 2011). A) Visual representation of the Triple Network Model (TNM), comprising SN, FPN and DMN functional networks (a representative slice shown for all three). Such model highlights the top-down communication from SN to FPN and SN to DMN (directed solid arrows) and hence, communication between FPN and DMN (dashed horizontal line) mediated by SN. B) Cohort average functional connectome highlighting functional couplings of the TNM. C) Distribution of the average functional couplings for region pairs within (full lines in B) and between three *a priori* functional networks (dashed outlines in B). Vertical dashed line illustrates the division between positive and negative functional couplings.

Relying on the TNM framework, we focused on the functional connectivity within and between SN, FPN, and DMN functional networks, which reduced the number of brain regions from 332 to 145 and highlighted neural communication patterns specific to these networks (see Figure 2B). Using a proposed 17 network solution (Thomas Yeo et al., 2011), the three functional networks were determined as follows: SN corresponds to the Ventral Attention Network A and Ventral Attention Network B, FPN is comprised of Control A, Control B and Control C, while the Default Mode Network includes Default A, Default B, and Default C. Additionally, and guided by the TNM, we excluded direct functional interactions between FPN and DMN, allowing PLS to only model communication between the SN and the FPN, and between the SN and the DMN. This strategy relies on the TNM framework assumption that the SN influences both the FPN and DMN (V. Menon, 2011) and its relationship to specific AUD characteristics.

The phenotypic domain consists of eight variables: three demographic and five AUD-related (see Table 2). The connectivity domain is characterized by the vectorized functional connectivity profiles for each participant (upper triangle of FC matrix excluding the main diagonal). The statistical relationships between both domains capture underlying associations between participant phenotypes and rsFC.

### 2.8 Regularized Partial Least Squares (rPLS)

As with many other learning methods (Tian & Zhang, 2022), regularization can also be applied in PLS. Here, we simultaneously regularized both domains to shrink the coefficients in both domains concurrently. PLS regularization is a data-driven feature subset selection strategy that adds reliability by preventing overfitting and contributes to the interpretability of the results (Tibshirani, 1996). The connectivity domain comprises brain region pairwise coupling information and is represented by high dimensional vectors. As learning methods are ill-posed when presented with high-dimensional data (i.e., more variables than samples) (Trunk, 1979), regularization addresses high dimensionality and provides fine-grained results (increased coefficient specificity) that eases interpretability.

Our goal was to identify functional couplings that play an important role in the overall relationship between the phenotypic characteristics and brain connectivity patterns. We computed both standard (i.e. non-regularized) and regularized PLS solutions using the Matlab implementation described in (Monteiro et al., 2016). This rPLS implementation provides flexibility for different regularization levels for each data domain by using two independent regularization parameters (one per domain). The range of possible values for each regularization parameter, $\lambda_{D}$, is $1\leq \lambda_{D}\leq \sqrt{\left|D\right|}$ where |D| represents the number of variables in the respective domain (maximal regularization attained when $\lambda_{D}=1$). For the connectivity domain, we opted for a regularization level that preserves 50% of the original (non-sparse) solution to gain specificity on the connectivity domain. For the phenotypic domain, we selected the smallest regularization that restricts the first component to a maximum of 3 features (see Figures S7 and S8). The orthogonality between components is achieved via a deflation procedure in which new components are iteratively computed on the residuals of the previous ones (Monteiro et al., 2016).

### 2.9 Network interaction significance testing

We identified significant functional edges by applying a null model to assess the significance of each network interaction provided by the rPLS (connectivity coefficients). For each component, we constructed a null model by randomly shuffling its connectivity coefficients and compared this randomized connectivity profile with the true coefficients (see Contreras et al., 2017). Network interactions with total contribution (sum of absolute value of coefficients) higher than the corresponding null model ensemble (99th percentile; 1,000 null model runs) were considered significant. This approach allowed us to identify high-level connectivity patterns significantly represented in the connectivity domain coefficients of each rPLS component (see Figure S9).

### 2.10 Within cohort stability of rPLS components

Leave-one-out cross validation (LOOCV) assessed stability by leaving out one sample of the data at a time and training the model on the remaining samples. This process was repeated for each sample in the dataset. The model’s stability is evaluated using the ensemble of resulting models and subsequent outputs (coefficients), which provides a more robust description of its behavior (Efron, 1979; Stone, 1977). We computed 55 leave-one-out PLS iterations, equal to the number of participants in the sample. Our LOOCV analyses included the coefficients distribution for each phenotypic variable, as well as the coefficients standard deviation for each functional coupling in the connectivity domain.

## 3. Results

### 3.1. Associations between AUD-related characteristics and resting-state functional connectivity

**Figure 3.**
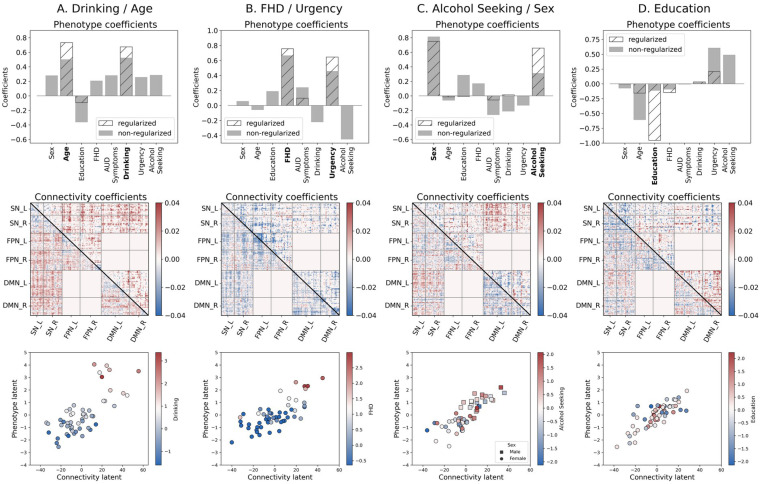
First four PLS components (non-regularized; gray bars, regularized (${\text{λ}}_{C}=49.0$, ${\text{λ}}_{P}=1.5$); hatched bars in the phenotype coefficients). Based on the TNM assumptions, we excluded the interactions between FPN and DMN (excluded blocks have all zeros in the Connectivity coefficient matrices). A) ***Drinking/Age*** component is dominated by Drinking and Age variables. B) ***Family History/Urgency*** component includes both FHD and Urgency, while AUD symptom count makes only a minor contribution. C) ***Alcohol Seeking/Sex*** component is mostly associated with alcohol seeking and Sex. D) ***Education*** component is predominantly associated with Education. Bottom row shows the latent associations between the two domains (Connectivity and Phenotype) as identified by PLS with the colored gradient representing the most prominent phenotypic variables in each component.

We used PLS to uncover associations of the eight phenotypic variables and rsFC patterns within a priori functional networks (FPN, SN, and DMN) and network interactions modeled by the TNM (Table 2). We assessed the goodness of fit for each component based on the covariance score and subsequently on the percentage of covariance with respect to the maximum (occurring, by definition, in the first component; see Figure S6). For each orthogonal component, PLS produces two coefficients sets, one for each domain (that is, AUD-related characteristics and connectivity). Each pair set describes a multivariate association between the domains and a different overall association. Figure 3 summarizes the associations between the two domains and the respective coefficients for each of the four analyzed components.

The connectivity set of coefficients obtained with PLS involves the entire connectivity domain of the TNM (that is, all edges potentially participate in the association). To uncover the most relevant functional couplings participating in the association, we used a regularized version of PLS (rPLS: Monteiro et al., 2016) as detailed in the Methods section. This resulted in regularization parameters $\lambda_{C}=49.0$ and $\lambda_{P}=1.5$ for the connectivity and phenotypic domains respectively for all PLS components (see Figures S7 and S8).

The results are illustrated by Figure 3. Given the predominant characteristics of the phenotypic domain, we named the components as follows:

- The *Drinking/Age* component denotes that, in this cohort, older participants with high drinking (first principal component of recent and lifetime drinking variables) have an increased between-network interactions of the SN with both FPN and DMN and decreased within network connectivity for all three networks.
- The *FHD/Urgency* component denotes that, in this cohort, participants with high family history density and high urgency have a decreased communication between the SN and FPN, as well as decreased within-network interactions of all three functional networks.
- The *Alcohol Seeking/Sex* component indicates that, in this cohort, males with high alcohol seeking behavior (PCA of willingness to work for alcohol across sessions) have an increased cross communication between the SN and DMN and decreased communication within the DMN.
- Lastly, the *Education* component is largely driven by years of education and does not involve any AUD trait. Hence, the subsequent analyses focus on the first three rPLS components.

### 3.2 Stability analysis of the rPLS components.

**Figure 4.**
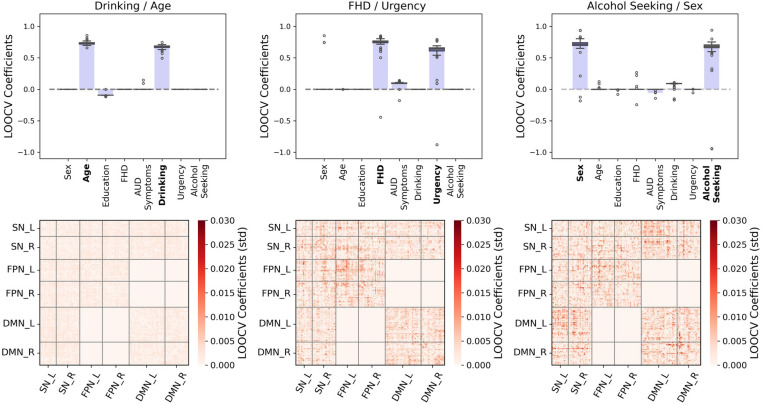
Leave-one-out cross validation of rPLS results (LOOCV; N=55) for the regularized PLS components. Top row: variability of the phenotypic characteristic coefficients across the LOOCV runs (boxplots) and full cohort coefficients for reference (purple bars). Bottom row: variability (standard deviation) of coefficients associated with each functional coupling along the LOOCV runs for each component. Interactions between FPN and DMN are excluded from the analysis following the assumptions of the TNM.

Variability across iterations was evaluated for each component and coefficient profile. LOOCV results (55 runs) show that the rPLS solution pattern that includes all participants is preserved across iterations (Figure 4). Specifically, the average effect of the dominant phenotypic variables (box plots) is well above zero and centered around the estimated coefficients in the regularized solution for the full cohort. The standard deviation of the connectivity coefficients is small for all components, where the Drinking/Age component shows the highest stability.

### 3.3 Neural substrates of the PLS components

To investigate the contributions of individual brain regions in each PLS component, we determined positive and negative strengths for each region. Briefly, given a connectivity matrix (here a connectivity coefficients matrix), the positive strength of a region is the sum of all positive coefficients in its row, whereas negative strength is the sum of all the row’s negative coefficients (Fornito et al., 2016). In our case, strength was obtained for each component, similar to other functional connectivity decomposition methods (Amico et al., 2017). Positive strength summarizes the direct association between an entire region’s connectivity profile and the phenotypic domain, whereas a negative strength summarizes the corresponding inverse association.

**Figure 5.**
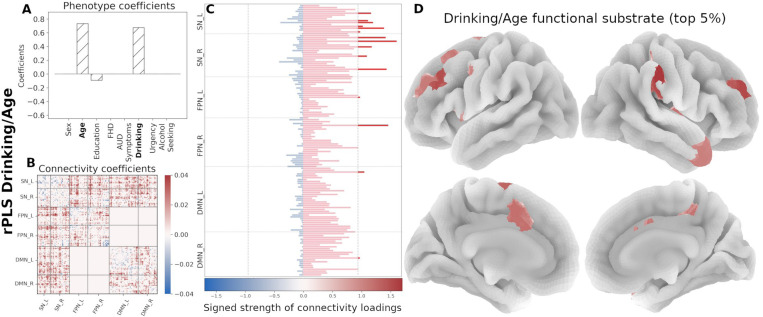
Neural substrate of ***Drinking/Age***. (A) Phenotype coefficients show Drinking and Age as the dominant factors (both with positive coefficients). (B) Connectivity coefficients show mainly increased interactions between functional networks. (C) Signed strength of connectivity coefficients with the contribution for each region. The gray line indicates the 95-percentile of strength distribution. (D) Brain rendering of the functional substrate for the top 5% regions with highest signed strength (regions above the threshold in panel C). Color gradient indicates the relative strength per region.

**Figure 6.**
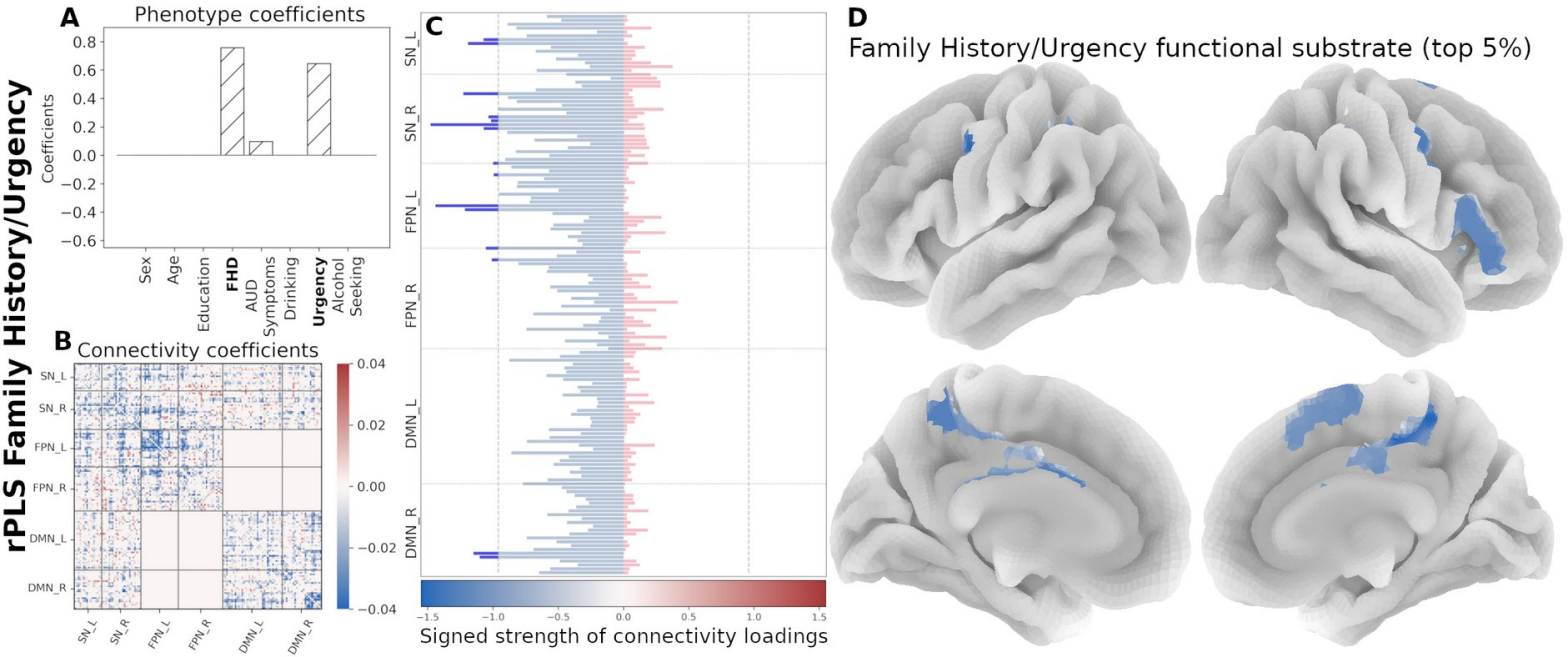
Neural substrate of *FHD/Urgency*. (A) Phenotype coefficients are dominated by FHD and Urgency, both with positive contributions. (B) Connectivity coefficients are marked by decreased interactions. (C) Signed strength of connectivity coefficients with the contribution for each region. The gray line indicates the 95-percentile of strength distribution. (D) Brain rendering of the functional substrate for the top 5% regions with highest signed strength (regions above the threshold in panel C). Color gradient indicates the relative strength per region.

**Figure 7.**
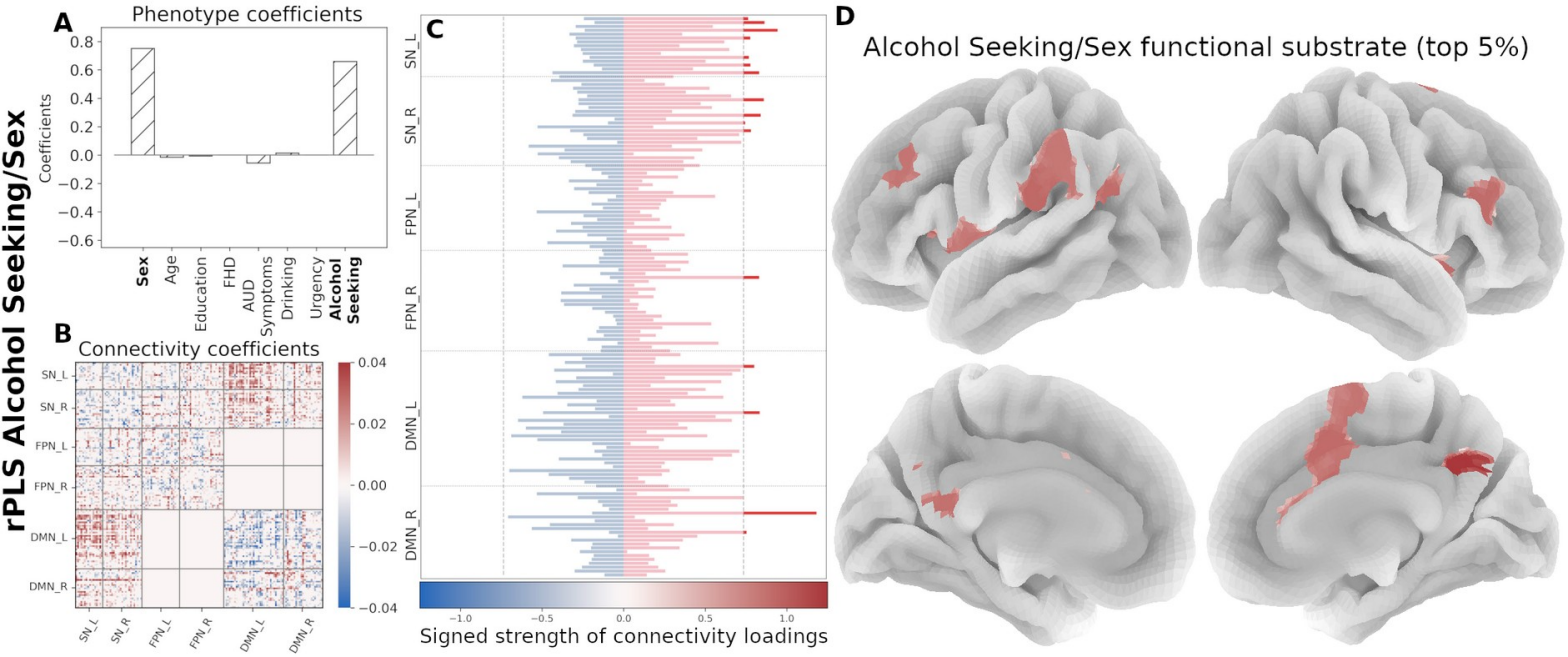
Neural substrate of *Alcohol Seeking/Sex*. (A) Phenotype coefficients are dominated by alcohol seeking (willingness to work for alcohol; positive coefficient) and age. (B) Connectivity coefficients in this component show increased contributions. (C) Signed strength of connectivity coefficients with the contribution for each region. The gray line indicates the 95-percentile of strength distribution. (D) Brain rendering of the functional substrate displaying the top 5% regions with highest signed strength (regions above the threshold in panel C). Color gradient indicates the relative strength per region.

Next, we organized the signed strength profile comprising all 145 regions by functional network membership and identified the top contributing regions (top 5%, resulting in 15 regions per component). In the Drinking/Age component, the strength profile shows primarily regions with increased connectivity, mainly within the SN and the FPN (Figure 5C). The *FHD/Urgency* component profile is marked by decreased functional connectivity in the SN and the FPN (Figure 6C). The strength profile for the *Alcohol Seeking/Sex* component includes regions with increased connectivity distributed between the SN and the DMN (Figures 7C).

**Figure 8.**
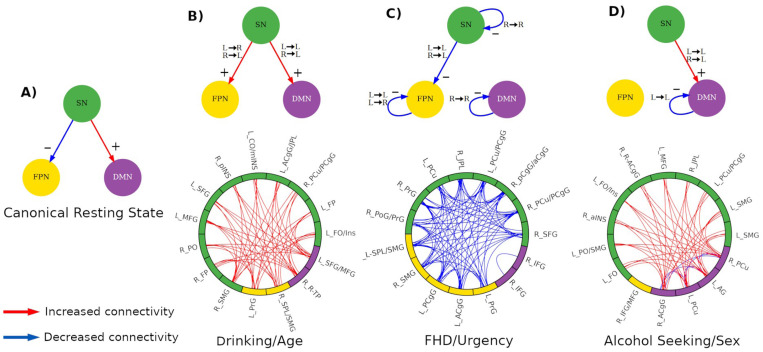
Schematic representation of the neural circuits underlying each component under the TNM and their association with AUD factors. **(A)** Canonical resting-state brain communication proposed by the TNM. **(B)** Drinking and Age are associated with an increased communication from SN to both FPN and DMN. **(C)** FHD and Urgency are associated with a decreased connectivity from SN to FPN and decreased within-connectivity in both SN and FPN. **(D)** Alcohol seeking and Sex (being male) is associated with an increased connectivity from SN to DMN and decreased within-connectivity in the DMN. Abbreviations: salience network (SN), frontoparietal network (FPN), default mode network (DMN), left hemisphere (L), right hemisphere (R). (B-D) Note that the sign (color) of the network interactions is inferred from the rPLS results whereas the directionality is imposed by the TNM model. The directionality of the black arrows (hemisphere specificity) follows the network directionality assumed by the TNM.

In the normative canonical resting-state connectivity circuit (under the TNM assumptions) the SN suppresses the FPN while enhancing activity in the DMN (Figure 8A). Using this canonical TNM circuit as a reference, we can summarize the results of each PLS component schematically by representing the main connectivity effects involving the three *a priori* functional networks (see Methods section and Figure S9). These diagrams capture distinct aspects of the relation between functional connectivity of these networks and AUD-related characteristics that represent high-level concurrent communication mechanisms involving SN, FPN and DMN (Figure 8). Here, the sign of the interactions is inferred from the rPLS results whereas the directionality is imposed by the TNM model.

The *Drinking/Age* circuit shows an increased communication of the SN with both FPN and DMN which, in combination with the TNM model, indicates a top-down regulation mechanism mediated by the SN. The left hemisphere of SN communicates with the left hemisphere of the FPN and the left hemisphere of the DMN while the right SN communicates with the left hemisphere of both FPN and DMN. The *FHD/Urgency* circuit is marked by decreased associations of the SN and the left FPN, as well as decreased communication within the right SN, left FPN and right DMN. Notably, communication between SN and DMN does not feature. The *Alcohol Seeking/Sex* circuit shows again findings consistent with a top-down structure, with increased communication between SN and the left DMN, while the communication within left DMN is decreased. PLS components are marked by specific connectivity patterns involving different subsets of brain regions; however, region contributions are not exclusive to a single component. Noteworthy is that components associated with drinking behavior (*Drinking/Age* and *Alcohol Seeking/Sex)* share regions in the left hemisphere of the SN, most prominently within the prefrontal and insulo-opercular (overlapping regions are shown in Figure S10 and listed in Table S1)

## 4. Discussion

In this work, we characterized the neural substrates and communication patterns associated with AUD-related characteristics at network and regional levels under a TNM framework, which hypothesizes that the SN mediates and recruits neural resources from the FPN and the DMN (V. Menon, 2011; Sridharan et al., 2008). Alterations in this three-network circuit appear in psychopathological conditions (Elsayed M, 2024; B. Menon, 2019; V. Menon, 2011), but the specific communication changes remain unclear. By combining the TNM, a data-driven analytical approach, resting state fMRI, and experimental design incorporating intravenous alcohol seeking paradigm in a sample of heavy drinkers, we uncovered specific concurrent communication mechanisms between the FPN, SN, and DMN resting-state functional networks as they relate to different AUD-associated characteristics spanning drinking, family history of AUD, AUD symptom count, urgency, biological sex, age, and laboratory-based alcohol self-administration. We found three major groups of AUD characteristics related to connectivity patterns in the TNM circuit: a Drinking/Age component, an FHD/Urgency component, and an Alcohol Seeking/Sex component. These findings indicate that AUD phenotypic features are associated with different interactions of the functional networks involved in the TNM. It is noteworthy that AUD symptom count was not a prominent factor in any of the rPLS components. Our analysis extends previous work showing AUD-related alterations in predefined regions as a function of AUD (Elsayed M, 2024; Suk et al., 2021) by including inter-relationships between networks within the TNM, and as a function of AUD-related characteristics and risk factors.

The SN, FPN and DMN are involved in a wide variety of cognitive and emotional processes and their coordination is thought to properly allocate neural resources in response to endogenous and exogenous demands. In turn, a range of psychiatric disorders are thought to affect these networks (Boehm et al., 2014; Rai et al., 2021; Stern et al., 2012; Suk et al., 2021). Findings of altered functional connectivity between these three networks in AUD and risky drinking include increased connectivity between the FPN and DMN (Suk et al., 2021), high resting-state connectivity in the FPN (Sousa et al., 2019), and abnormalities in the SN (Galandra et al., 2018; Suk et al., 2021).

The first component, the *Drinking/Age* component, is marked by increased communication between the SN and both the FPN and the DMN (see Figure 8), suggesting potentially increased top-down control. Aging positively contributes to this association, as well as drinking, which reflects both recent and lifetime drinking consumption patterns (Table 2). The observation that these two variables track together may be indicative of persistent and ongoing drinking patterns in this sample of heavy drinkers. The neural mechanism in this component suggests that the SN affects both the FPN and the DMN, which is consistent with previous findings (Suk et al., 2021) that FPN and the DMN are simultaneously active during resting state. In healthy individuals at rest, the FPN and the DMN are negatively correlated, suggesting functional specificity and a mechanism to coordinate neural resources in response to internal and external cognitive demands (Deming et al., 2023; V. Menon, 2011). Positive correlation between the FPN and DMN during rest may indicate a lack of coordination, reduced functional independence, suboptimal neural responses to cognitive demands, and compensatory mechanisms to sustain brain functionality. Examples of these abnormal brain configurations and their association with multiple psychopathological disorders are reported in numerous studies (Boehm et al., 2014; He et al., 2021; Rai et al., 2021; Stern et al., 2012; Suk et al., 2021; Zhang & Volkow, 2019).

The *FHD/Urgency* component was marked by decreased communication between the SN and the FPN and decreased within-network communication in all three networks. Decreased functional interactions are a marker of disassociation between brain processes, a signature of automatic processes that do not require cognitive effort (Finc et al., 2017; Kitzbichler et al., 2011). For urgency, this may be indicative of a neural configuration suited for automatic (non-regulated) predisposition towards impulsive behavior, as urgency is theorized to reflect lower top-down and higher bottom-up processing (Cyders & Smith, 2008; G. T. Smith & Cyders, 2016). Several studies have largely supported dysregulation between these two systems as related to urgency (see Um et al., 2019; Zorrilla & Koob, 2019). In addition, this component is characterized by decreased within-network connectivity in all three networks, which has been linked to impaired executive dysfunction, emotion regulation, and risk assessment (Clark et al., 2008; Grodin et al., 2017; Singer et al., 2009). Explanations for reduced connectivity within the FPN include weak intrinsic connectivity within its nodes and constrained access to salience stimuli from the SN (Weiland et al., 2014).

Recent evidence suggests that SN dysfunction affects cognitive performance in individuals with AUD, but this impairment is largely mediated through the FPN (Rawls et al., 2021). SN dysfunction also causes impaired mapping of salience events and disrupted balance of appropriate neural resources (V. Menon, 2011), consequences of which include weak emotion regulation and a lack of cognitive control (V. Menon, 2011; Seeley et al., 2007; Zilverstand et al., 2018). Reduced connectivity within the SN has been observed in individuals after periods of acute alcohol consumption (Gorka et al., 2018), is linked to inability to restraint subjective urges (Sullivan et al., 2013), and is a predictor for future relapse (Camchong et al., 2022; Kohno et al., 2017). Our findings contrast with one resting state study that found increased within-network connectivity in the SN, orbitofrontal cortex, and the DMN and increased between-network connectivity as a function of negative urgency in those with AUD (Zhu et al., 2017). In our work, the neural circuit related to urgency also reflects FHD, suggesting that automatic engagement in rash behavior may be also related to genetic risk factors for AUD, as shown in former studies (Dick et al., 2010; Stephenson et al., 2023).

The *Alcohol Seeking/Sex* component was characterized by increased communication between the SN and DMN and reduced communication within the left DMN. Communication between the DMN and the SN is enhanced during the withdrawal phases in addiction (Zhang & Volkow, 2019). Altered connectivity between the DMN and cortical regions associated with memory and emotion regulation is critical for compulsive drug seeking despite adverse consequences. Decreased connectivity within the DMN has been associated with several substance use disorders, (Vergara et al., 2017) including AUD (Müller-Oehring et al., 2015), which is reflective of reduced self-awareness and rumination during alcohol abstinence. In a recent study (Muller et al., 2024) assessing network configurations during active alcohol approach, the DMN was found to be an important network for information integration, suggesting a possible configural state that facilitates greater intensity of alcohol seeking. Sex is an important and well documented AUD risk factor (Becker et al., 2012; Flores-Bonilla & Richardson, 2020; George B. Richardson & Brian B. Boutwell, 2020; Plawecki et al., 2018). Its presence in the component could represent effects from sex alone or in combination with appetitive effort for alcohol.

Combining participant phenotypes in a clinically meaningful manner helps to understand how gradients of AUD risk relate to the TNM circuit. Each TNM circuit (Figure 8) characterizes the neural substrate representative of AUD risk factors. By comparing these with the “canonical TNM circuit” (for healthy controls in resting-state; Figure 8A), we begin to interpret how large-scale mechanisms might form signatures of these factors. For example, while both FHD and drinking are related in the population, the data here suggest that these factors involve very different signatures of communication within the TNM circuit. Data such as these may therefore facilitate targeted interventions aimed at specific clinical features.

In contrast to region specific seed-based analyses or testing univariate associations with a single phenotype, we assessed whether the communication patterns within and between TNM networks are associated with AUD-related characteristics. Using a combination of experimental design, theoretical model, and data-driven approaches is a core strength of this work. This methodology allows us to understand how complex interactions involving multiple AUD-related characteristics shape the communication patterns between *a priori* functional networks. In addition, this work extends the current application of the TNM to a sample with heavy alcohol use and provides an interpretative framework approach to better understand TNM alterations specific to different psychopathological disorders.

LOOCV results show that the identified relationships are stable (the pattern is preserved across iterations), and the contributions (coefficients) of individual variables and brain connectivity patterns are preserved overall (Figure 4). The variables with the greatest contributions are on average the same, which means that the associations captured by the components are stable within the cohort and less likely to be driven by specific participants.

Our findings and interpretations have several limitations that should be considered. First, the modest sample size may impact the replicability and robustness of our findings. Second, cross-sectional data and the statistical method (PLS) preclude causal interpretations of the inferred associations and interactions between networks. Third, our sample is by design restricted to participants who endorse heavy alcohol use, with about 60% meeting criteria for AUD, which may impact the generalizability of our findings. That said, AUD symptom count was itself not related to the connectivity patterns. Finally, the analysis of resting-state data was not complemented by the task fMRI assessments that could target specific AUD-relevant brain regions (e.g., reward system) and behaviors (e.g., alcohol cue-response, working for alcohol reward, etc.).

The long-term goal of this work is to contribute to the design and test targeted neural interventions to mitigate and prevent AUD. Although neuromodulation approaches are becoming feasible to reduce AUD symptoms, such as craving and alcohol use (Alba-Ferrara et al., 2014; Mehta & Parasuraman, 2013), there is large heterogeneity in effects across individuals, suggesting that more foundational work is needed to determine the most effective mechanisms of action. This study starts to lay that foundation by beginning to identify functional connectivity patterns that may serve as biomarkers of combinations of phenotypes. Subsequent steps would be to replicate and extend these findings in a larger data set, especially one with more variability in alcohol use and demographic characteristics. Additionally, identifying specific region-to-region interactions would better determine optimal targets for neuromodulation, while relating the functional role of these interactions to AUD-related characteristics can maximize treatment selection and efficiency. Causal discovery methods and experimental trials of the network association described in this work will help to disentail top-down from bottom-up neural process in AUD.

## 5. Conclusion

We combined theory- and data-driven approaches to document underlying neural substrates characterizing drinking, family history of AUD, AUD symptom count, urgency, and alcohol seeking, all factors known to impart risk for AUD. Focusing on the TNM, this study provides an approach for a comprehensive characterization of the neural components underlying AUD, revealing how the brain networks unfold into concurrent characteristic-specific configurations. This study demonstrates the utility of data-driven approaches in uncovering associations between resting-state functional substrates and phenotypic characteristics that could aid in the identification, development, and testing of novel treatment targets across preclinical and clinical models.

## Supplementary Material

Supplement 1

## Figures and Tables

**Table 1.** Participant phenotypes. FHD: Biological Family History of Alcoholism (Stoltenberg & Dd, 1998). CAT: Constant Attention Task (Plawecki et al., 2013).

Participant characteristics (N=55)			
	**Mean (SD)**	**Range**	**Units**

Sex	24 M, 31 F		
Age	32.18 (9.99)	21 – 55	Years
Education	15.40 (2.08)	11 – 20	Years
Family History of AUD Density (FHD)	0.07 (0.12)	0 – 0.42	Density
AUD Symptoms	2.5 (2.36)	0 – 10	Scalar
**Drinks per Drinking Day (TLFB_DDD_)**	5.26 (4.10)	1.7 – 26	Standard drinks
**Drinking Days per Week (TLFB_DDW_)**	1.72 (1.72)	0 – 7	Days
**Drinks per Week (TLFB_DW_)**	21.20 (26.78)	3.2 – 182	Standard drinks
**Greatest Number of Drinks in a Single Day (TLFB_GDD_)**	10.44 (6.45)	3 – 32	Standard drinks
**Lifetime total alcohol consumption (LDH_KG_)**	183.56 (348.10)	5.3 – 2185.87	Kilograms
Positive Urgency (Pur)	6.55 (2.54)	4 – 12	Scalar
Negative Urgency (Nur)	8.07 (2.64)	4 – 15	Scalar
Neutral Condition Cumulative Work for Water (N_cww)	200.73 (203.60)	0 – 771	Number of completed CAT Trials
Neutral Condition Cumulative Work for Alcohol (N_cwa)	254.84 (216.52)	1 – 707	Number of completed CAT Trials
Aversive Condition Cumulative Work for Water (A_cww)	180.89 (173.79)	0 – 616	Number of completed CAT Trials
Aversive Condition Cumulative Work for Alcohol (A_cwa)	270.65 (195.78)	1 – 708	Number of completed CAT Trials

**Table 2.** Participant characteristics used in the partial least square analysis as a phenotypic domain.

Participant Phenotypic Characteristics (N=55, female=31)	
	Description
Sex	Self-reported
Age	Chronological age (years)
Education	Amount of education (years)
FHD	Biological family history density of alcohol use disorder
AUD Symptoms	AUD symptom count form the SSAGA interview
Drinking	1^st^ PCA (TLFB_DDD_, TLFB_DDW_, TLFB_DW_, TLFB_GDD_, LDH_KG_)
Urgency	1^st^ PCA (Positive Urgency, Negative Urgency)
Alcohol seeking	1^st^ PCA (Aversive Condition Alcohol Preference, Neutral Condition Alcohol Preference)
