# Supplementary material for: Resting State Functional Connectivity Patterns Associate with Alcohol Use Disorder Characteristics: Insights from the Triple Network Model
Source: ArXiv. 2025 Apr 8:arXiv:2504.06199v1. Preprint. [Version 1] (PMC12036437)
Supplement: Supplement 1 [file NIHPP2504.06199v1-supplement-1.pdf]

Supplementary Material

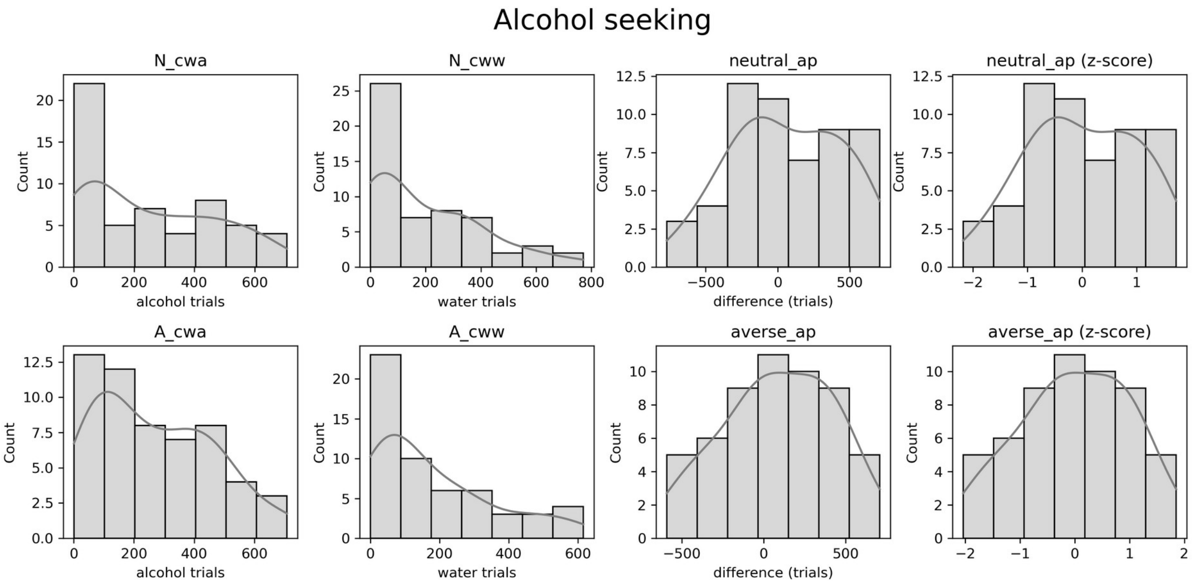

Figure S1. Cumulative work (number of trials) for alcohol and water in self-administrated sessions. A_cwa: cumulative work for alcohol in aversive session. A_cww: cumulative work for water in aversive session. N_cwa: cumulative work for alcohol in neutral session. N_cww: cumulative work for water in neutral session. The difference between alcohol and water in each session provides contrast reflecting the excess between each reward

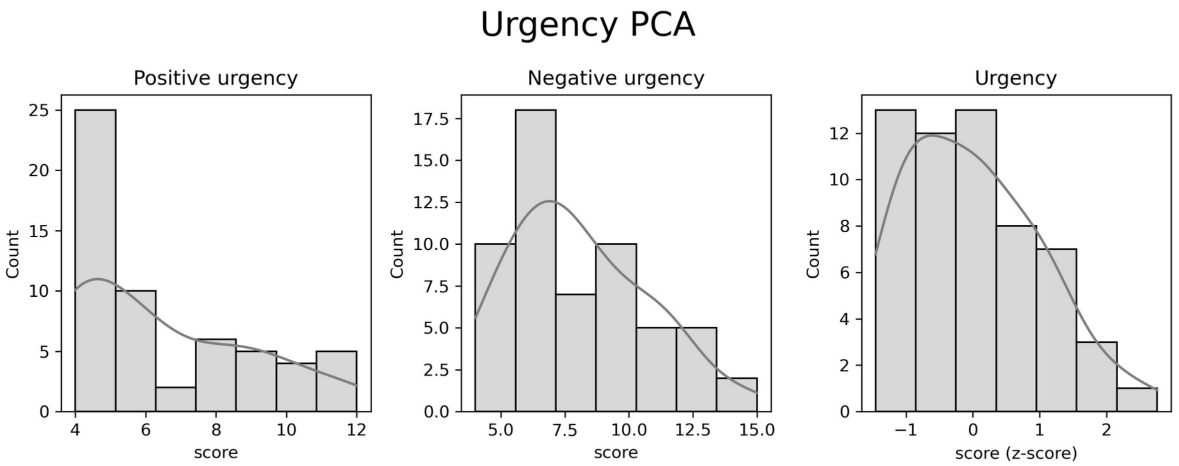

Figure S2. Two impulsivity factors, positive and negative urgency are combined into a single urgency variable using PCA (first component explained 75% of the variance).

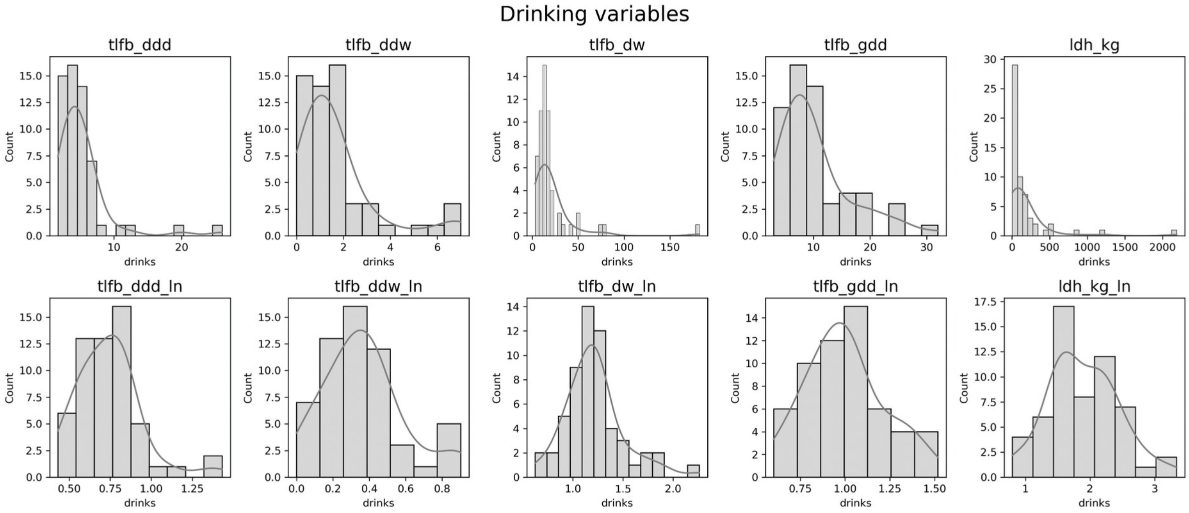

Figure S3. Drinking related variables capture recent and long-term drinking patterns. tlfb_ddd: drinks per drinking day, tlfb_ddw: drinks per drinking week, tlfb_dw:, tlfb_gdd: maximum drinks per drinking day, ldh_kg: lifetime drinking history measured in kilograms. The original variables (measured in drinking units) presented skewed distributions and were logarithmically. The logarithmic variables are used as input to PCA to compute the Drinking variable comprising drinking patterns.

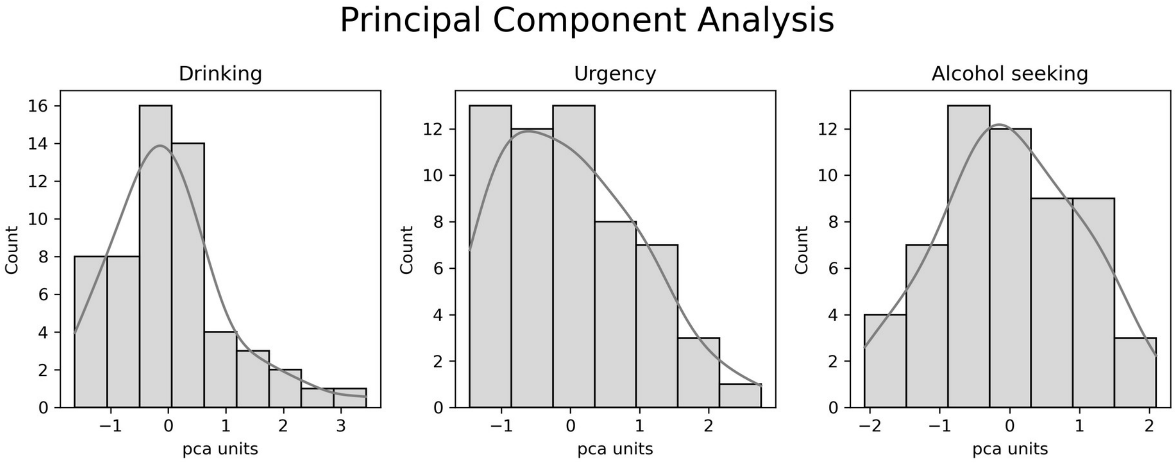

Figure S4. The first principal component was obtained for each of the following three sets of variables. **Drinking:** first component of ltfb_ddd, ltfb_ddw, ltfb_dw, ltfb_gdd, ldh_kg (70% explained variance). **Urgency:** first component of positive and negative urgency (75% explained variance) of. **Alcohol seeking:** first component of aversive_ap and neutral_ap (74% explained variance).

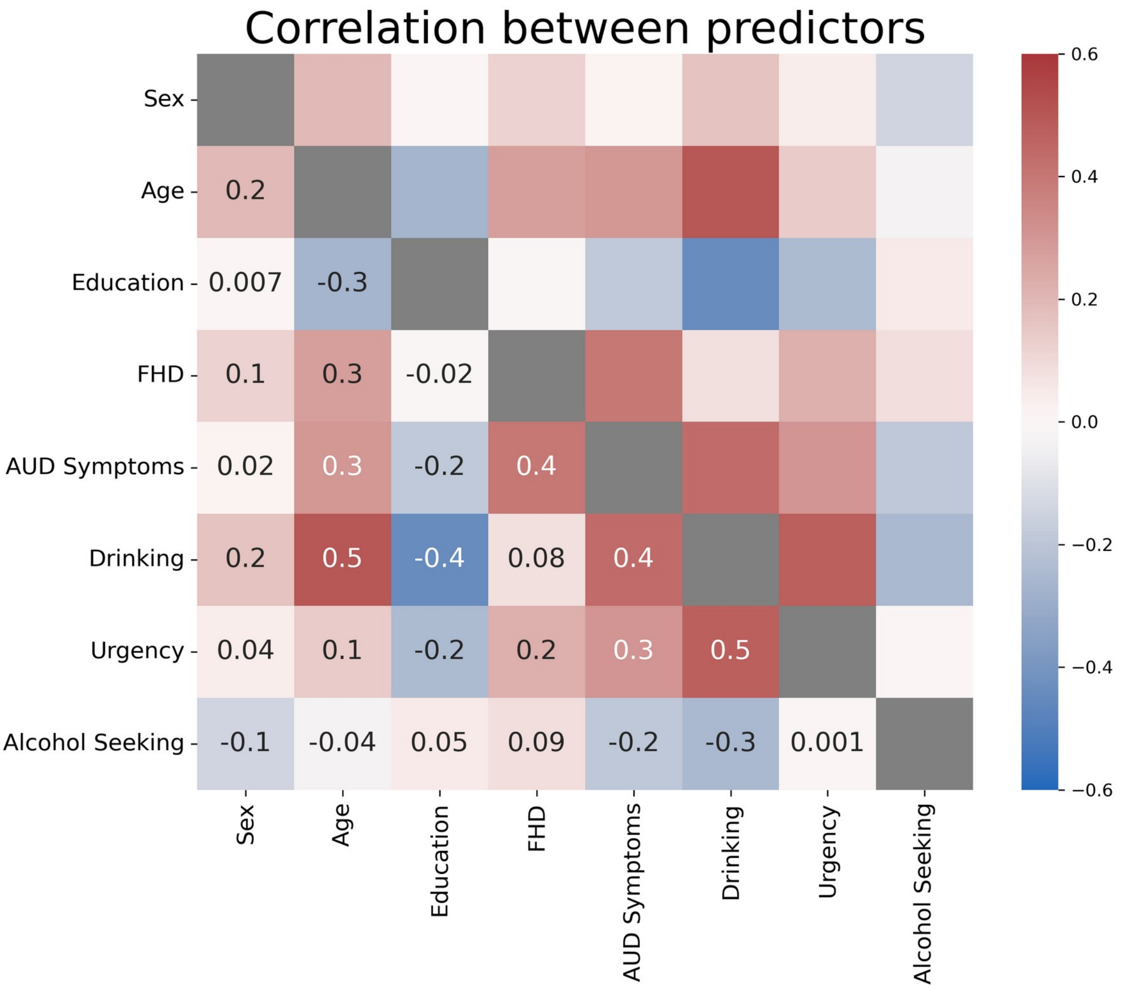

Figure S5. Pairwise correlations between the variables spanning the phenotype domain used as input to the Partial Least Square model.

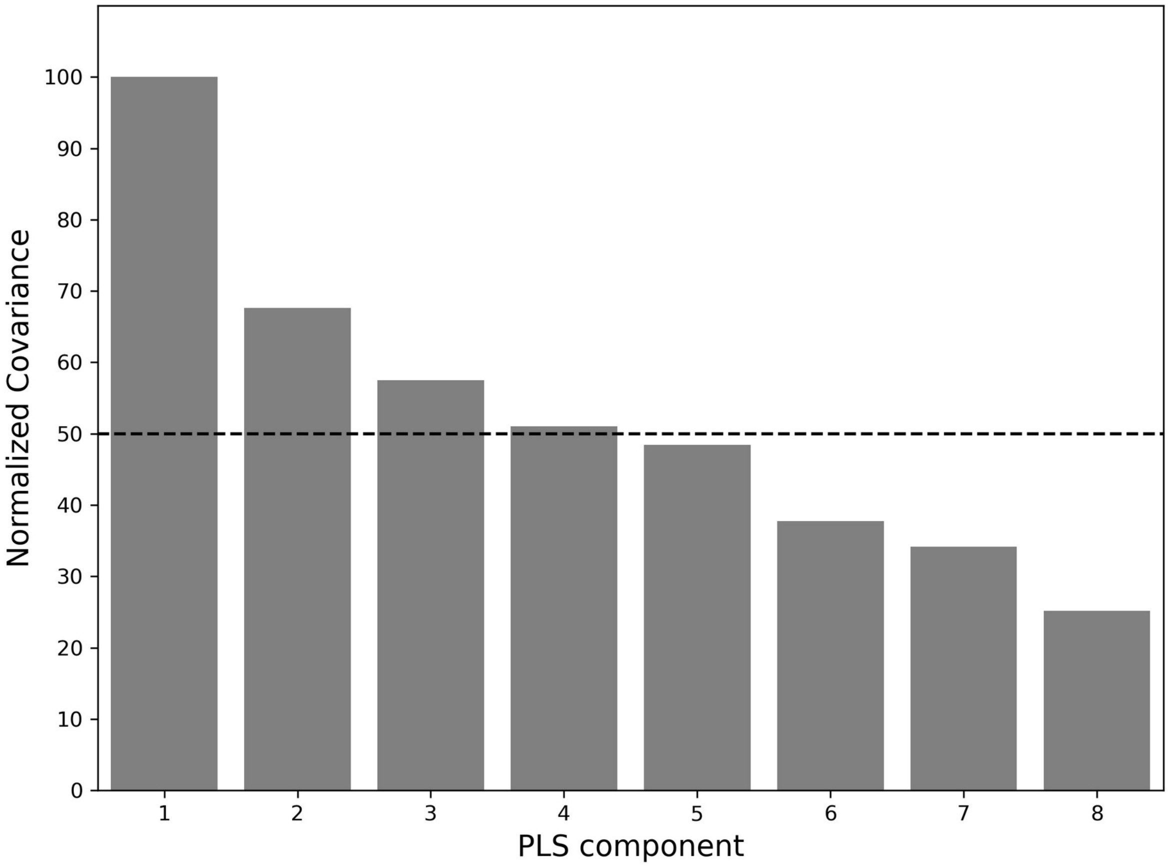

Figure S6. Covariance associated with each of the eight PLS components, normalized with respect to the maximum covariance (Component 1: 100%). A threshold criterion of 50% for the relative covariance retains the first four PLS components for subsequent analyses.

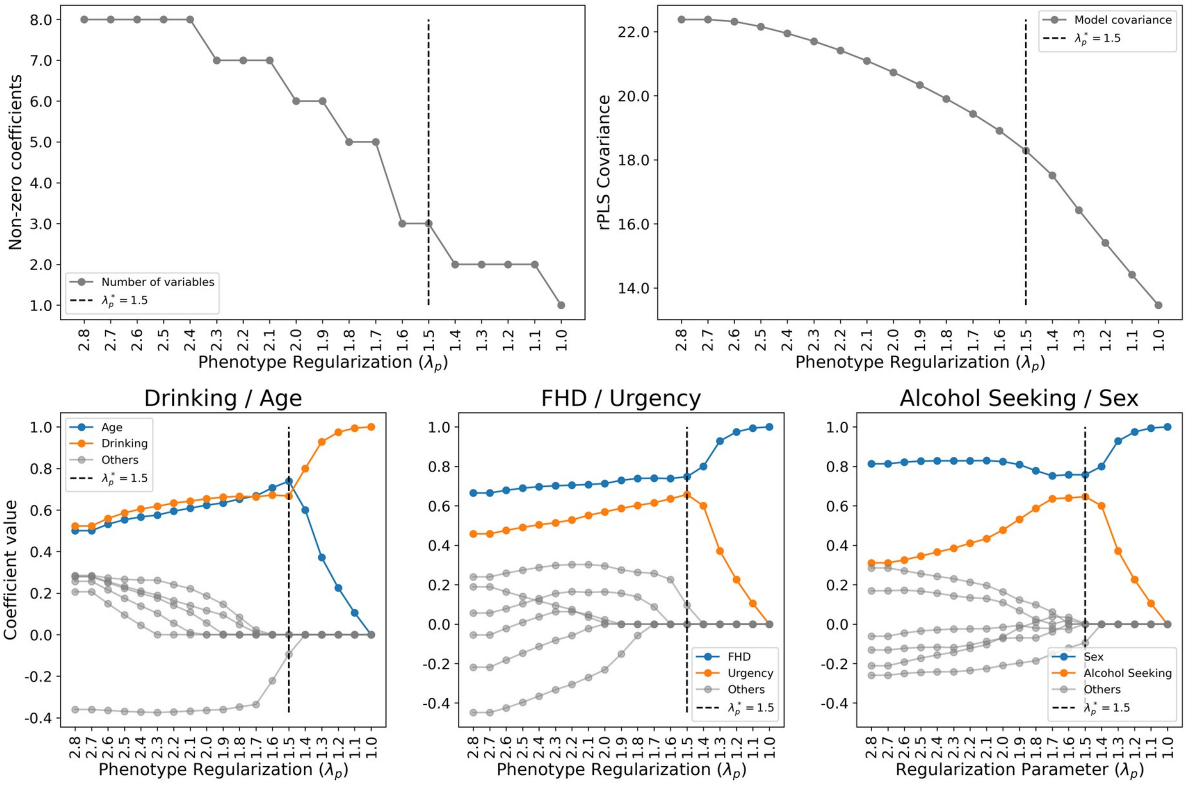

Figure S7. The regularization parameter for the phenotypic domain (1.5 indicated by the red horizontal line) was selected based on the number non-zero coefficients in component 1.

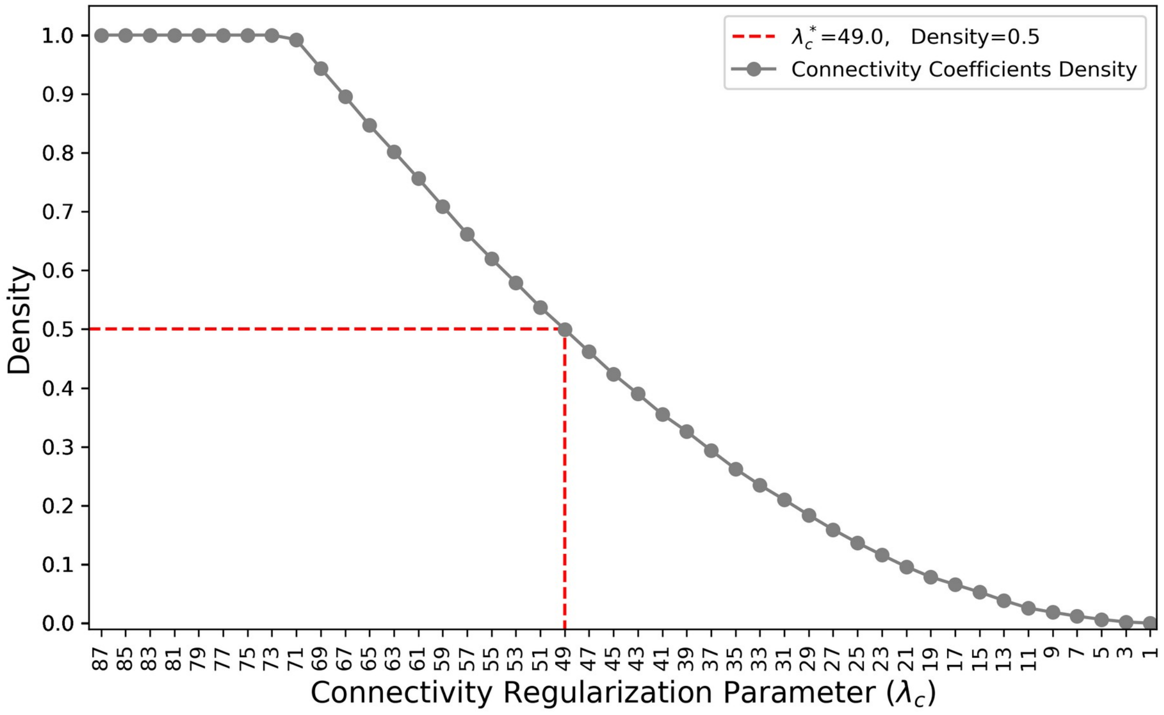

Figure S8. The Partial Least Squares regularization parameter (lambda) was set to the value 49.0. At this value, the density of the connectivity solution (number of functional edges participating in the solution) is 50%.

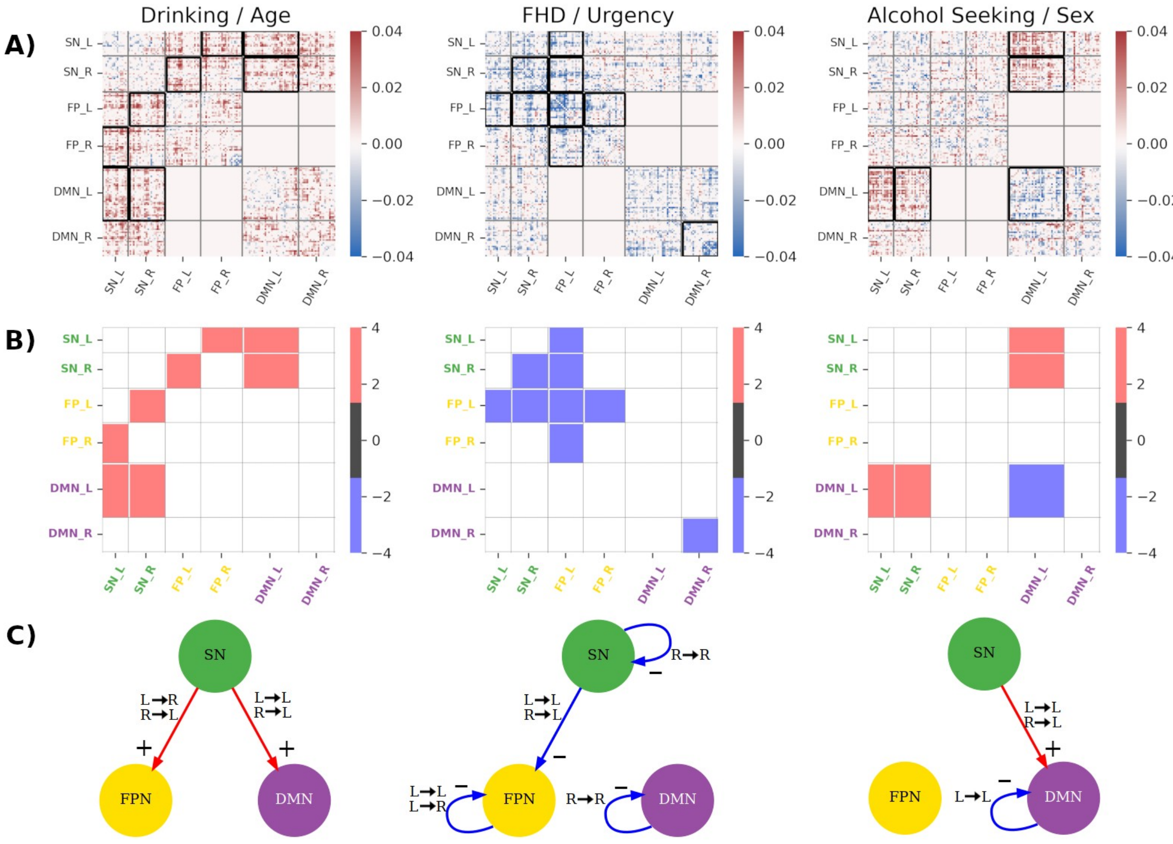

Figure S9. Statistical significance of network interactions for each rPLS component.

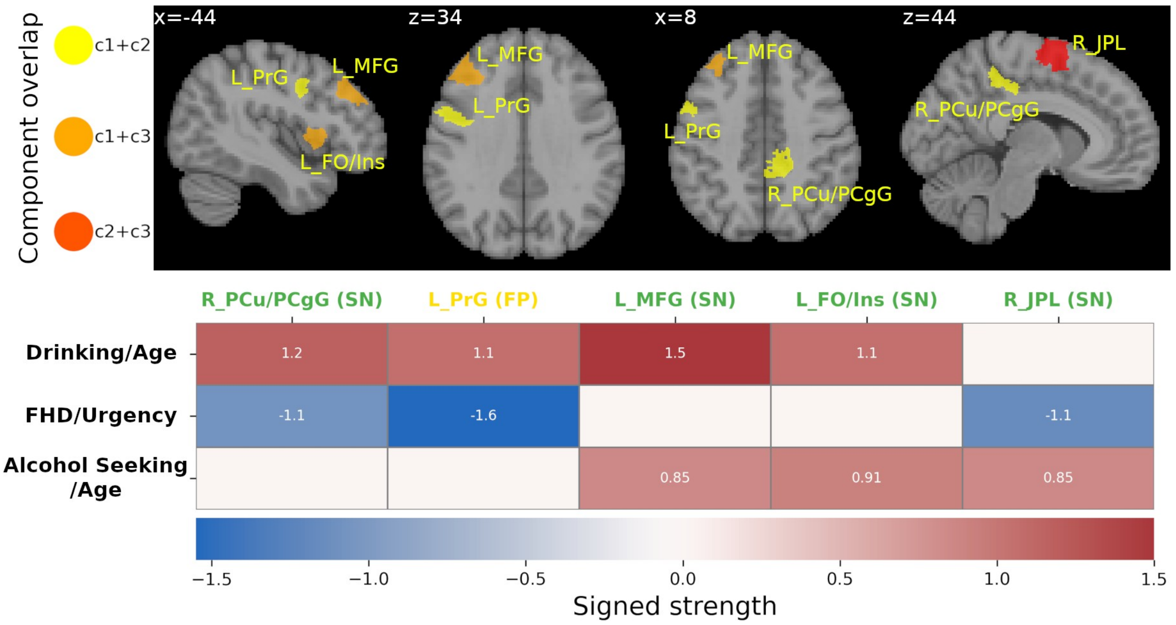

Figure S10. Overlapping regions between rPLS components.

Drinking/Age Component

Network	Hemisphere	Schaefer 300 index	Schaefer 300 region	Description	Sign	Strength
SN	R	214	SalVentAttnA_ParOper_4	SMG	+	1.73
FPN	R	248	ContA_IPS_3	SPL/SMG	+	1.58
SN	R	229	SalVentAttnB_PFCl_2	FP	+	1.54
SN	R	212	SalVentAttnA_ParOper_2	PO	+	1.52
SN	L	76	SalVentAttnB_PFCl_2	MFG	+	1.50
SN	L	73	SalVentAttnA_FrMed_2	SFG	+	1.29
SN	R	217	SalVentAttnA_Ins_2	pINS	+	1.27
SN	L	68	SalVentAttnA_Ins_3	CO/mINS	+	1.25
SN	L	72	SalVentAttnA_FrMed_1	ACgG/JPL	+	1.20
SN	R	222	SalVentAttnA_ParMed_2	PCu/PCgG	+	1.17
DMN	L	113	DefaultA_PFCd_2	SFG/MFG	+	1.13
SN	L	75	SalVentAttnB_PFCl_1	FP	+	1.10
FPN	L	99	ContA_PFCl_4	PrG	+	1.06
DMN	R	284	DefaultB_AntTemp_1	R-TP	+	1.05
SN	L	78	SalVentAttnB_Ins_2	FO/Ins	+	1.05

Family History/Urgency Component

SN	R	223	SalVentAttnA_ParMed_3	PoG/PrG	-	1.59
FPN	L	99	ContA_PFCl_4	PrG	-	1.55
SN	R	215	SalVentAttnA_PrC_1	PrG	-	1.31
FPN	L	100	ContA_Cingm_1	ACgG	-	1.30
SN	L	71	SalVentAttnA_ParMed_2	PCu	-	1.28
DMN	R	288	DefaultB_PFCv_2	IFG	-	1.23
DMN	R	289	DefaultB_PFCv_3	IFG	-	1.18
SN	R	224	SalVentAttnA_FrMed_2	JPL	-	1.15
SN	L	70	SalVentAttnA_ParMed_1	PCu/PCgG	-	1.14
FPN	L	110	ContC_Cingp_1	PCgG	-	1.13
SN	R	221	SalVentAttnA_ParMed_1	pCgG/aCgG	-	1.11
FPN	R	247	ContA_IPS_2	SMG	-	1.08
SN	R	222	SalVentAttnA_ParMed_2	PCu/PCgG	-	1.08
SN	R	233	SalVentAttnB_PFCmp_2	SFG	-	1.07
FPN	L	91	ContA_IPS_2	L-SPL/SMG	-	1.07

Alcohol Seeking/Sex Component

DMN	R	277	DefaultA_pCunPCC_3	PCu	+	1.54
SN	L	67	SalVentAttnA_Ins_2	FO	+	1.16
SN	R	216	SalVentAttnA_Ins_1	aINS	+	1.13
SN	L	65	SalVentAttnA_ParOper_2	PO/SMG	+	0.91
SN	R	220	SalVentAttnA_FrMed_1	R-ACgG	+	0.85
SN	L	78	SalVentAttnB_Ins_2	FO/Ins	+	0.81
DMN	L	126	DefaultB_IPL_1	AG	+	0.74
FPN	R	251	ContA_PFCl_2	IFG/MFG	+	0.71
DMN	L	114	DefaultA_pCunPCC_1	PCu	+	0.71
SN	R	224	SalVentAttnA_FrMed_2	JPL	+	0.7
SN	L	76	SalVentAttnB_PFCl_2	MFG	+	0.68
SN	L	69	SalVentAttnA_FrOper_1	PCu/PCgG	-	0.99
SN	L	74	SalVentAttnB_IPL_1	SMG	-	0.79
SN	L	64	SalVentAttnA_ParOper_1	SMG	-	0.68
DMN	R	282	DefaultA_PFCm_5	ACgG	-	0.66

Education Component

SN	R	221	SalVentAttnA_ParMed_1	pCgG/aCgG	+	1.22
DMN	R	289	DefaultB_PFCv_3	IFG	+	1.12
DMN	L	141	DefaultC_Rsp_1	pCgG	+	1.01

DMN	L	112	DefaultA_PFCd_1	SFG	+	1.00
DMN	L	132	DefaultB_PFCd_5	SFG	+	0.92
SN	R	222	SalVentAttnA_ParMed_2	PCu/PCgG	+	0.92
DMN	R	274	DefaultA_PFCd_1	MFG	+	0.91
SN	R	226	SalVentAttnB_IPL_1	FP	-	1.45
SN	R	219	SalVentAttnA_FrOper_1	CO	-	1.41
SN	L	73	SalVentAttnA_FrMed_2	SFG	-	1.31
SN	R	216	SalVentAttnA_Ins_1	aINS	-	1.17
SN	R	218	SalVentAttnA_Ins_3	CO/mINS	-	0.91
SN	R	221	SalVentAttnA_ParMed_1	pCgG/aCgG	-	0.91
SN	R	217	SalVentAttnA_Ins_2	pINS	-	0.90
SN	R	224	SalVentAttnA_FrMed_2	JPL	-	0.84

Table S1 Top brain regions per component (top 5%). Index refers to Schaefer 300 parcellation (Schaefer et al., 2018) ACgG: Anterior Cingulate Gyrus, ACgG/JPL: Anterior Cingulate Gyrus/Juxtapositional Lobule, aINS: Ventral Anterior Insula, aINS/OFC: Anterior Insula/Orbitofrontal Cortex, CO/mINS: Central Operculum/Middle Insular Cortex, FO: Frontal Operculum, FO/OFC: Frontal Operculum/Lateral Orbitofrontal Cortex, FrP: Frontal Pole, IFG: Inferior Frontal Gyrus, IFG/MFG: Inferior/Middle Frontal Gyrus, INS: Insula, JPL: Juxtapositional Lobule Cortex, LOC: Lateral Occipital Cortex (superior), MFG: Middle Frontal Gyrus, MFG/IFG: Middle/Inferior Frontal Gyrus, MTG: Middle Temporal Gyrus, PCgG: Posterior Cingulate Cortex (retrosplenial), pCgG/aCgG: Cingulate Gyrus (posterior and anterior), PCu: Precuneus (anterior/dorsal), PCu/PCgG: Precuneus/Posterior Cingulate Gyrus, pINS: Ventral Posterior Insula, PO: Parietal Operculum, PoG/PrG: Postcentral/Precentral Gyrus (medial), extends into posterior Cingulate Gyrus (anteriorly) and Precuenus (posteriorly), PrG: Precentral Gyrus, PrG/IFG: Precentral/Inferior Frontal Gyrus, SFG: Superior Frontal Gyrus/Paracingulate Gyrus, SFG/MFG: Superior/Middle Frontal Gyrus, SMG: Supramarginal Gyrus (anterior), SPL/SMG: Superior Parietal Lobule/Supramarginal Gyrus (posterior)
